# Cribriform pattern 4/intraductal carcinoma of the prostate and persistent prostate‐specific antigen after radical prostatectomy

**DOI:** 10.1002/bco2.367

**Published:** 2024-05-15

**Authors:** Takeshi Sasaki, Ikuo Kobayashi, Katsunori Uchida, Shinichiro Higashi, Satoru Masui, Kouhei Nishikawa, Toyonori Tsuzuki, Masatoshi Watanabe, Naoto Sassa, Takahiro Inoue

**Affiliations:** ^1^ Department of Nephro‐Urologic Surgery and Andrology, Graduate School of Medicine Mie University Tsu Japan; ^2^ Department of Urology Aichi Medical University Nagakute Japan; ^3^ Department of Oncologic Pathology, Graduate School of Medicine Mie University Tsu Japan; ^4^ Department of Surgical Pathology Aichi Medical University Nagakute Japan

**Keywords:** cribriform, intraductal carcinoma, persistent PSA, prostate cancer, radical prostatectomy

## Abstract

**Objectives:**

The objective of this study is to identify the effect of cribriform pattern 4 carcinoma/intraductal carcinoma of the prostate (CC/IDCP) on persistent prostate‐specific antigen (PSA) levels after robot‐assisted radical prostatectomy (RARP) in patients with localized prostate cancer (PCa).

**Patients and Methods:**

This retrospective study included 730 consecutive patients with localized PCa who underwent RARP at Mie University (*n* = 392) and Aichi Medical University (*n* = 338) between 2015 and 2021. Patients with clinically metastatic PCa (cN1 and cM1) and those who received neoadjuvant and/or adjuvant therapy before biochemical recurrence were excluded. We evaluated the effects of CC/IDCP on persistent PSA levels after RARP. Persistent PSA was defined as PSA level ≥0.2 ng/mL at 1 month postoperatively and consecutively thereafter. Using factors from logistic regression analysis, models were developed to predict persistent PSA levels.

**Results:**

Approximately 6.3% (*n* = 46) of the patients had persistent PSA levels. Patients with biopsy CC/IDCP (bCC/IDCP) and pathological CC/IDCP (pCC/IDCP) based on RARP specimens were 11.6% (85/730) and 36.5% (267/730), respectively. Multivariate analysis of the prediction of persistent PSA levels using preoperative factors revealed that PSA density, percentage of positive cancer cores, biopsy grade group and bCC/IDCP were independent prognostic factors. Furthermore, multivariate analysis of the prediction of persistent PSA levels using postoperative factors, excluding pN1, revealed that pathological grade group, pCC/IDCP, seminal vesicle invasion and lymphovascular invasion were independent prognostic factors. In the receiver operating characteristic curve analysis for predicting persistent PSA after RARP, areas under the receiver operating characteristic curve for the model with preoperative factors, postoperative factors, including pN1, and postoperative factors, excluding pN1, were 0.827, 0.833 and 0.834, respectively.

**Conclusions:**

bCC/IDCP predicted persistent PSA after RARP in the overall population, while pCC/IDCP predicted persistent PSA only when the pN1 population was excluded. This may be useful for predicting susceptible patients with worse outcomes.

## 1 INTRODUCTION

Persistent prostate‐specific antigen (PSA) was reported in cases in which the postoperative PSA nadir did not reach PSA < 0.1–0.2 ng/mL at 4–8 weeks after surgery.^1^, ^2^ Patients with persistent PSA levels after radical prostatectomy (RP) were assigned to the poor prognosis group. Preisser et al. demonstrated that at 15 years after RP, metastasis‐free survival (MFS), overall survival and cancer‐specific survival were 53.0% versus 93.2%, 64.7% versus 82.1% and 75.5% versus 96.2% (all *P* < 0.001) for persistent versus undetectable PSA, respectively.^3^ Therefore, guidelines established from a collaboration among the American Urology Association, American Society for Radiation Oncology and Society of Urologic Oncology recommend androgen‐deprivation therapy, in addition to salvage radiation therapy, for patients with biochemical recurrence (BCR) after RP who have persistent PSA.^4^ In a recent review article, the incidence of persistent PSA ranged between 3.1% and 34.6%, with a median of 11.0%.^2^ Previous studies have shown that several clinicopathological variables, such as the D'Amico risk (high risk vs. medium and low risk), postoperative International Society of Urologic Pathology (ISUP) grade group ≥4, pathological T stage ≥pT3, extraprostatic extension (EPE), seminal vesicle invasion (SVI), positive surgical margins (PSMs) and lymphovascular invasion (LVI), were independently associated with persistent PSA after robot‐assisted RP (RARP).^2^

Recently, systematic reviews have revealed that cribriform pattern 4 carcinoma/intraductal carcinoma of the prostate (CC/IDCP) is a predictor of adverse pathological and clinical outcomes in RP cohorts.^5^, ^6^ The presence of CC/IDCP based on RP specimens was associated with EPE, SVI, lymph node metastasis, BCR, distant metastasis and cancer‐specific mortality (CSM).^6^ The ISUP indicated that CC, confluent sheets of contiguous malignant epithelial cells with multiple glandular lumens, should be regarded as part of the spectrum of the Gleason grade 4 pattern, as well as glomeruloid, fused and poorly formed glands.^6^ In contrast, IDCP is characterized by malignant cell growth within pre‐existing prostatic ducts.^7^ Because CC and IDCP are histologically similar and some cases cannot be differentiated by haematoxylin and eosin staining without immunohistochemical staining of basal cells (34BetaE12 or p63),^8^ many studies have examined CC/IDCP combined.^6^, ^7^, ^9^

Although several studies have evaluated the association between persistent PSA levels after RP and clinicopathological variables, there have been no reports on the association between CC/IDCP and persistent PSA levels after RARP in patients with localized prostate cancer (PCa). This study aimed to identify the effects of CC/IDCP on persistent PSA levels after RARP.

## 2 PATIENTS AND METHODS

### 2.1 Study participants

A retrospective multicentre cohort study was conducted among patients with PCa who underwent RARP at two Japanese institutions between February 2015 and December 2021. Patients who received neoadjuvant and/or adjuvant therapy before BCR were excluded. The study was approved by the Institutional Review Boards of Mie University (approval number: 2022‐167) and Aichi Medical University School of Medicine (approval number: 2022‐102). Primary endpoint was prognostic effect of CC/IDCP on persistent PSA levels after RARP.

For preoperative stage classification, all the patients underwent whole‐body computed tomography, pelvic magnetic resonance imaging and bone scans, and the biopsy ISUP grade group and biopsy CC/IDCP (bCC/IDCP) were recorded. Patients with clinically metastatic cN1 and/or cM1 PCa were excluded from the study. PSA density was determined by preoperative volume estimation using transurethral ultrasonography. Pathological T and N stages of the surgical specimens were recorded, as well as pathological ISUP grade group (pGG), EPE, SVI, positive surgical margins, LVI and pathological CC/IDCP (pCC/IDCP). Patients with incomplete pathological data were excluded. All tumours were staged according to the American Joint Committee on Cancer Eighth Edition Cancer Staging Manual. All the patients in this study underwent RARP. The presence or absence of pelvic lymph node dissection, its extent and use of a nerve‐sparing approach were determined by a surgeon or policies of each institution.

### 2.2 Pathological analysis

All prostate biopsy and prostatectomy specimens were evaluated, according to the ISUP 2014 guidelines.^10^ Pathologists shaved the prostatic apex vertically against the prostatic urethra, cut a conical section of the bladder neck margin from the specimen and sectioned it vertically. For the remaining prostate tissue, complete sections were prepared at 3 mm intervals along a plane perpendicular to the urethral axis. Prostate biopsy and prostatectomy specimens were reviewed by experienced genitourinary pathologists, U. K. (Mie University Hospital) and T. T. (Aichi Medical University Hospital), who were blinded to the clinical outcomes.

### 2.3 Follow‐up schedule

After the surgery, all the patients were evaluated for serum PSA levels at 1 month postoperatively and thereafter were evaluated at 1–3 month intervals. According to the Japanese Urological Association guidelines,^11^ the criteria for persistent PSA is defined as a patient whose PSA had not decreased to <0.2 ng/mL at 1 month postoperatively and thereafter had never decrease to <0.2 ng/mL.

### 2.4 Statistical analysis

Univariate and multivariate analyses were performed using logistic regression. Only variables found to be significant in the univariate analysis (*P* < 0.05) were included in the multivariate analysis. Using factors from logistic regression analysis, models were developed to predict persistent PSA levels after RARP. The contribution of each variable to persistent PSA prediction (standardized coefficient: beta [β]‐coefficient) was determined, and a formula for calculating the persistent PSA prediction score (multiple regression formula) was created.^12^ To keep the scores close to integers and intuitive for the user, all β‐coefficients were standardized with the lowest having a value of 1. Individual risk scores were obtained by summing up the risk factors. Statistical analyses were performed using IBM SPSS Statistics for Windows version 28 (IBM Corp., Armonk, NY, USA). Statistical significance level was set at *P* < 0.05.

## 3 RESULTS

A total of 730 consecutive patients with localized PCa who underwent RARP from the Mie University Hospital (*n* = 392) and Aichi Medical University Hospital (*n* = 338) were enrolled in the study. Clinical characteristics of the patients in this cohort are presented in Table 1. Patients with persistent PSA levels were 6.3% (*n* = 46). Positive bCC/IDCP and pCC/IDCP based on RARP specimens were 11.6% (85/730) and 36.5% (267/730), respectively. Of the 85 patients with positive bCC/IDCP, 60 (70.5%) had positive pCC/IDCP based on RARP specimens. In contrast, of the 267 patients with positive pCC/IDCP based on RARP specimens, 60 (22.4%) had positive bCC/IDCP. Three year MFS overall survival rates were 89.9% and 98.8% for persistent and without persistent PSA levels, respectively (Figure S1A; *P* < 0.001). Three year castration‐resistant PCa‐free survival rates were 93.0% and 99.1% for persistent and without persistent PSA levels, respectively (Figure S1B; *P* = 0.003).

**TABLE 1:** Descriptive characteristics for 730 patients that underwent robot‐assisted radical prostatectomy (RARP).

Number of patients	730
Median follow‐up (range) (months)	33 (1–96)
NCCN criteria (%)	
Favourable risk	109 (15)
Intermediate risk	358 (49)
High risk and very high risk	263 (36)
Median age (range) (years at surgery)	68.5 (43–81)
Median initial PSA (range) (ng/mL)	7.2 (0.7–90.5)
Median initial PSAD (range) (ng/mL/cm^3^)	0.26 (0.03–5.36)
Median number of biopsy cores (range)	12 (4–70)
Clinical T stage at diagnosis (%)	
cT1/T2/T3a/T3b	133/541/52/4 (18/74.5/7/0.5)
Percentage of positive cancer cores (%)	33.3 (1.4–100)
Biopsy GG (%)	
1	152 (20.5)
2	209 (29)
3	136 (18.5)
4	122 (17)
5	111 (15)
Biopsy CC/IDCP (%)	
Negative/positive	645/85 (88.5/11.5)
Pathological T stage (%)	
pT2/T3a/T3b/4	501/157/71/1 (69/21.5/9.5/0.1)
Pathological GG (%)	
1	68 (9)
2	291 (40.5)
3	183 (25)
4	52 (7)
5	136 (18.5)
Pathological CC/IDCP based on RARP specimens (%)	
Negative/positive	463/267 (64/36)
Pathological N stage (%)	
pN0/N1/Nx (cN0)	542/25/163 (74/3.5/22.5)
Surgical margins (%)	
Negative/positive	507/223 (69.5/30.5)
EPE (%)	
Negative/positive/NA	465/229/36 (63.5/31.5/5)
SV invasion (%)	
Negative/positive	659/71 (90/10)
LVI (%)	
Negative/positive	650/80 (89/11)
Median number of resected lymph nodes (range)^a^	6.5 (0–40)
Nerve sparing (%)	
None/unilateral/bilateral	398/188/144 (54/26/20)

Abbreviations: CC/IDCP, cribriform pattern 4 carcinoma/intraductal carcinoma of the prostate; EPE, extraprostatic extension; GG, grade group; LVI, lymphovascular invasion; NA, not available; NCCN, National Comprehensive Cancer Network; PSA, prostatic‐specific antigen; PSAD, PSA density; SV, seminal vesicle.

^a^ Excluded 163 patients without pelvic lymph node dissection.

We first evaluated the preoperative predictors of persistent PSA levels after RARP (Table 2). Multivariate analysis revealed that PSA density (ng/mL/cm^3^) (≥0.5 vs. <0.5; odds ratio [OR], 3.38; 95% confidence interval [CI], 1.74–6.55; *P* < 0.001), percentage of positive cancer cores (%) (≥50 vs. <50; OR, 2.20; 95% CI, 1.13–4.28; *P* = 0.019), biopsy GG (≥4 vs. <4; OR, 4.60; 95% CI, 2.15–9.83; *P* < 0.001) and bCC/IDCP (yes vs. no; OR, 2.37; 95% CI, 1.14–4.90; *P* = 0.020) were independent prognostic factors.

**TABLE 2:** Univariate and multivariate analyses of preoperative factors predicting persistent prostatic‐specific antigen (PSA) after robot‐assisted radical prostatectomy (RARP) (*N* = 730).

Factors	Univariate	Multivariate
	Odds ratio [95% CI] (*P*‐value)	Odds ratio [95% CI] (*P*‐value)
Age (years) at surgery (<68 vs. ≥68)	1.10 [0.60–2.01]	—
	(*P* = 0.749)	
PSA levels (ng/mL) at surgery (≥20 vs. <20)	3.78 [1.64–8.71]	—
	(*P* = 0.002)	
PSAD (ng/mL/cm^3^) (≥0.5 vs. <0.5)	5.05 [2.74–9.32]	3.38 [1.74–6.55]
	(*P* < 0.001)	(*P* < 0.001)
Percentage of positive cancer cores (%) (≥50 vs. <50)	3.72 [2.01–6.86]	2.20 [1.13–4.28]
	(*P* < 0.001)	(*P* = 0.019)
Clinical T stage (≥3 vs. <3)	3.85 [1.79–8.25]	—
	(*P* < 0.001)	
Biopsy primary Gleason score 5 (yes vs. no)	3.35 [1.21–9.24]	—
	(*P* = 0.019)	
Biopsy GG (≥4 vs. <4)	6.95 [3.52–13.70]	4.60 [2.15–9.83]
	(*P* < 0.001)	(*P* < 0.001)
Biopsy CC/IDCP (yes vs. no)	5.31 [2.77–10.16]	2.37 [1.14–4.90]
	(*P* < 0.001)	(*P* = 0.020)

*Note*: All hazard ratios represent the ratios of the left‐hand‐side variables to the right‐hand‐side variables. Only the variables found to be significant in univariate analysis (*P* < 0.05) were entered into the multivariate analysis. For the results of the multivariate analysis, only selected variables (*P* < 0.05) were listed in the table; variables that were not selected were em dashed.

Abbreviations: CC/IDCP, cribriform pattern 4 carcinoma/intraductal carcinoma of the prostate; GG, grade group; PSAD, PSA density.

Next, we evaluated the postoperative factors based on RARP specimens as predictors of persistent PSA levels after RARP (Table 3). Model 1 selected clinically useful factors based on the univariate analysis results, including pathological N (pN) stage. Multivariate analysis revealed that pGG (≥4 vs. <4; OR, 3.66; 95% CI, 1.76–7.62; *P* < 0.001), SVI (yes vs. no; OR, 2.28; 95% CI, 1.00–5.16; *P* = 0.048) and pN (yes vs. no; OR, 5.44; 95% CI, 2.08–14.20; *P* < 0.001) were the independent prognostic factors. In Model 2, multivariate analysis was performed, excluding the pN factor, which implies locally metastatic cancer. Multivariate analysis revealed that pGG (≥4 vs. <4; OR, 3.94; 95% CI, 1.92–8.09; *P* < 0.001), pCC/IDCP (yes vs. no; OR, 2.29; 95% CI, 1.13–4.65; *P* = 0.021), SVI (yes vs. no; OR, 3.24; 95% CI, 1.49–7.02; *P* = 0.003) and LVI (yes vs. no; OR, 2.30; 95% CI, 1.05–5.02; *P* = 0.035) were independent prognostic factors.

**TABLE 3:** Univariate and multivariate analyses of postoperative factors predicting persistent prostatic‐specific antigen (PSA) after robot‐assisted radical prostatectomy (RARP) (*N* = 730).

	Univariate	Model 1 Multivariate	Model 2 Multivariate
Factors	Odds ratio [95% CI] (*P*‐value)	Odds ratio [95% CI] (*P*‐value)	Odds ratio [95% CI] (*P*‐value)
Pathological T stage (≥3 vs. <3)	7.10 [3.60–14.00]	2.58 [1.12–5.97]	—
	(*P* < 0.001)	(*P* = 0.026)	
Pathological primary Gleason score 5 based on RARP specimens (yes vs. no)	3.50 [1.26–9.69]	—	—
	(*P* = 0.016)		
Pathological GG based on RARP specimens (≥4 vs. <4)	7.73 [4.02–14.86]	3.66 [1.76–7.62]	3.94 [1.92–8.09]
	(*P* < 0.001)	(*P* < 0.001)	(*P* < 0.001)
Pathological CC/IDCP based on RARP specimens (yes vs. no)	4.36 [2.28–8.34]	—	2.29 [1.13–4.65]
	(*P* < 0.001)		(*P* = 0.021)
Nerve sparing (unilateral or bilateral vs. none)	0.35 [0.17–0.71]	—	—
	(*P* = 0.003)		
Surgical margins (positive vs. negative)	3.21 [1.75–5.89]	—	—
	(*P* < 0.001)		
EPE (positive vs. negative or NA)	5.65 [2.95–10.81]	—	—
	(*P* < 0.001)		
SV invasion (positive vs. negative)	8.55 [4.45–16.40]	2.28 [1.00–5.16]	3.24 [1.49–7.02]
	(*P* < 0.001)	(*P* = 0.048)	(*P* = 0.003)
LVI (positive vs. negative)	7.18 [3.77–13.67]	—	2.30 [1.05–5.02]
	(*P* < 0.001)		(*P* = 0.035)
Pathological N stage (1 vs. 0 or x)	14.73 [6.46–33.59]	5.44 [2.08–14.20]	Not included
	(*P* < 0.001)	(*P* < 0.001)	

*Note*: All hazard ratios represent the ratios of the left‐hand‐side variables to the right‐hand‐side variables. Only the variables found to be significant in univariate analysis (*P* < 0.05) were entered into the multivariate analysis. For the results of the multivariate analysis, only selected variables (*P* < 0.05) were listed in the table; variables that were not selected were em dashed.

Abbreviations: CC/IDCP, cribriform pattern 4 carcinoma/intraductal carcinoma of the prostate; EPE, extraprostatic extension; GG, grade group; LVI, lymphovascular invasion; NA; not available; SV, seminal vesicle.

**FIGURE 1:**
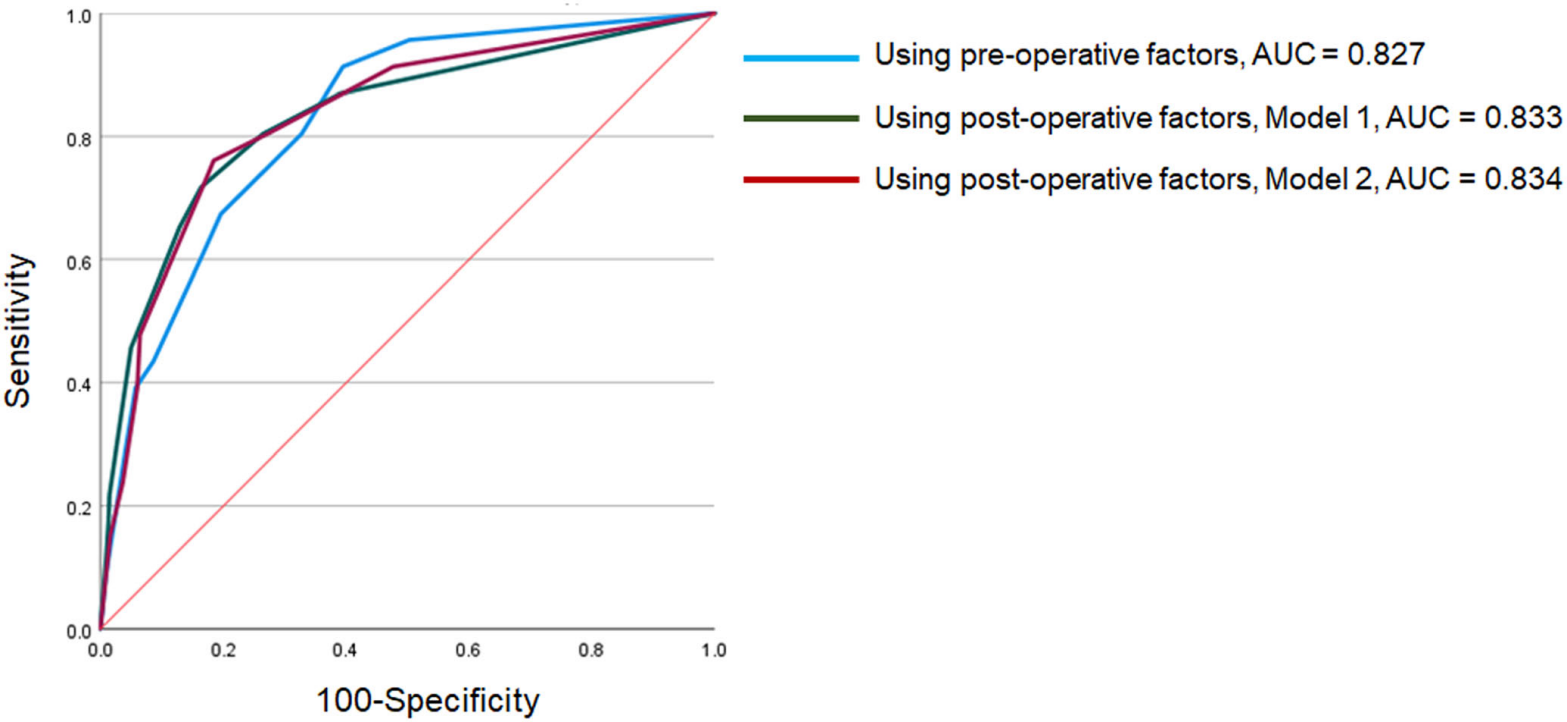
Receiver operating characteristic curve analysis was used to evaluate the predictive value of persistent prostate‐specific antigen after robot‐associated radical prostatectomy. The area under the curve (AUC) of persistent prostate‐specific antigen after robot‐associated radical prostatectomy using the model with preoperative factors, postoperative factors, including pN1, and postoperative factors, excluding pN1, were 0.827, 0.831 and 0.834, respectively.

Table 4 shows the risk assessment by scoring the factors for each model with preoperative factors and Models 1 and 2 with postoperative factors, all of which were standardized, with the lowest having a value of 1. In the receiver operating characteristic curve analysis for predicting persistent PSA after RARP, the areas under the receiver operating characteristic curve for the model with preoperative factors, postoperative factors, including pN1 (Model 1), and postoperative factors, excluding pN1 (Model 2), were 0.827, 0.833 and 0.834, respectively (Figure 1).

**TABLE 4:** Beta (β) coefficient estimation from the multivariate logistic regression analyses and scoring evaluation by risk factors.

A. Using preoperative factors		
Risk factors	β	Point
PSAD (ng/mL/cm^3^) (≥0.5)	1.218	1.5
Percentage of positive cancer cores (%) (≥50)	0.792	1
Biopsy GG (≥4)	1.527	2
Biopsy CC/IDCP (yes)	0.863	1

B. Using postoperative factors, Model 1		
Risk factors	β	Point
Pathological T stage (≥3)	0.951	1
Pathological GG based on RARP specimens (≥4)	1.300	1.5
SV invasion (positive)	0.824	1
Pathological stage N1	1.694	2

C. Using postoperative factors, Model 2		
Risk factors	β	Point
Pathological GG based on RARP specimens (≥4)	1.374	1.5
Pathological CC/IDCP based on RARP specimens (yes)	0.831	1
SV invasion (positive)	1.176	1.5
LVI (positive)	0.835	1

D. Risk category	
Risk category	Total score
Low risk	≥0 and <2
Intermediate risk	≥2 and <4
High risk	≥4

Abbreviations: CC/IDCP, cribriform pattern 4 carcinoma/intraductal carcinoma of the prostate; GG, grade group; LVI, lymphovascular invasion; PSAD, prostatic specific antigen density; RARP, robot‐assisted radical prostatectomy; SV, seminal vesicle.

**FIGURE 2:**
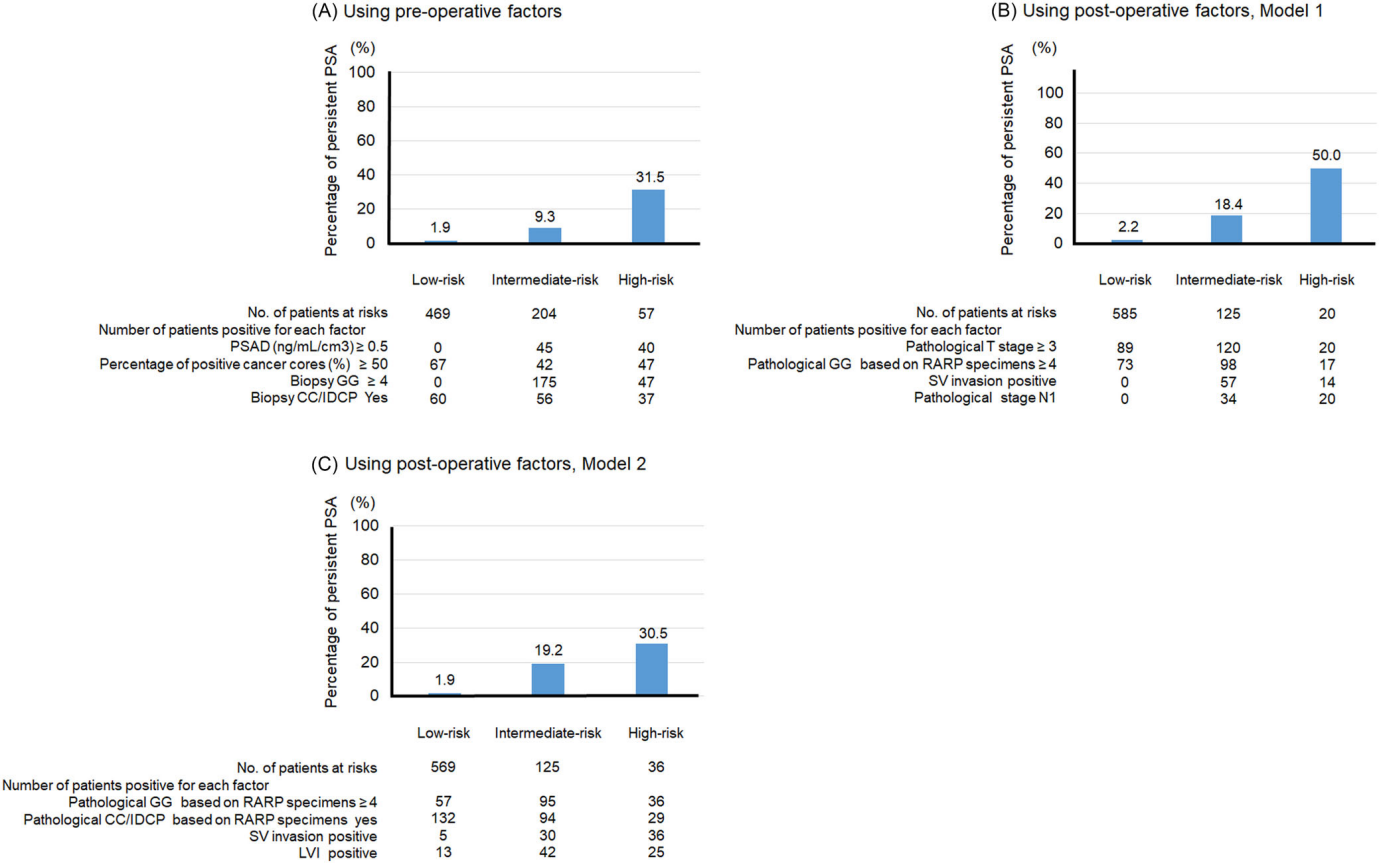
Distribution of scores and percentage of persistent prostate‐specific antigen (PSA) values after robot‐associated radical prostatectomy (RARP) for each model in low‐, intermediate‐, and high‐risk groups. (A) Using preoperative factors, (B) using postoperative factors, Model 1, and (C) using postoperative factors, Model 2. We defined a novel prognostic model in which patients with scores of ≥0 and <2 were defined as the low‐risk group, those with a total score of ≥2 and <4 were an the intermediate‐risk group, and those with a score of ≥4 were the high‐risk group. For all the models, the percentage of persistent PSA values after RARP was the highest in the high‐risk group. CC/IDCP, cribriform pattern 4 carcinoma/intraductal carcinoma of the prostate; GG, grade group; LVI, lymphovascular invasion; PSAD, PSA density; SV, seminal vesicle.

Based on these results, we developed a novel prognostic model in which patients with a total score of ≥0 and <2 were defined as a low‐risk group, those with a total score of ≥2 and <4 as an intermediate‐risk group, and those with a total score of ≥4 as a high‐risk group (Table 4). Figure 2 shows the distribution of scores and percentages of persistent PSA values after RARP for each model in the low‐, intermediate‐, and high‐risk groups. For all models, the percentage of persistent PSA values after RARP was the highest in the high‐risk group (Figure 2).

## 4 DISCUSSION

In our study, we evaluated the predictors of persistent PSA levels after RARP and found that PSA density, percentage of positive cancer cores, biopsy GG and bCC/IDCP were independent predictors of preoperative factors. Additionally, the significant predictors of postoperative factors, excluding pN1, were pGG, pCC/IDCP, SVI and LVI. We established new prognostic models using preoperative and postoperative factors, including CC/IDCP, that were effective in predicting persistent PSA levels. To the best of our knowledge, this is the first study to examine the effect of CC/IDCP on persistent PSA levels after RARP and show that bCC/IDCP predicted persistent PSA after RARP in the overall population, whereas pCC/IDCP predicted persistent PSA only when the pN1 population was excluded.

In our cohort, the PSA persistent rate was 6.3% (46/730 cases), which is slightly lower than previous reports, which we believe is due to the high cut‐off value of 0.2 ng/mL for persistent PSA, according to Japanese Urological Association criteria, or because RP was robot‐assisted. There are a number of reports that set the cut‐off value of persistent PSA at 0.1 ng/mL, and Micoogullari et al. reported that they reviewed cases with persistent PSA of 0.1–0.2 ng/mL and suggested salvage radiation therapy and androgen‐deprivation therapy for persistent PSA of ≥0.2 ng/mL.^13^ Rogers et al. reported that 47% of patients with persistent PSA levels developed distant metastases (median time to metastasis, 5.0 years; range 0.5–13 years).^14^ In our cohort, MFS and castration‐resistant PCa‐free survival were also significantly worse in the persistent PSA group than those in the nonpersistent PSA group.

In the analysis of preoperative factors for persistent PSA, four factors were found, two of which (biopsy ISUP grade group and percentage of positive cancer cores, with the exception of bCC/IDCP and PSA density) had already been included in the nomograms as preoperative tools for predicting RP outcomes.^15^ PSA density, which was better at detecting aggressive cancers than PSA^16^ in nomograms, is a significant factor in the prediction of persistent PSA. Finally, we identified bCC/IDCP as a new preoperative predictor of persistent PSA levels.

In the analysis of postoperative factors of persistent PSA, as pN1 is a strong prognostic factor for persistent PSA with local metastasis (PSA persistence rate as high as 26% for pN1^17^) (Model 1), we were interested in analysing the postoperative factors for persistent PSA in actual localized PCa (Model 2). We also examined whether CC/IDCP was a predictor of pN1 (Table S1) and found that CC/IDCP was a predictor of pN1 as in the previous report,^18^ suggesting that CC/IDCP might be a confounding factor when including pN1; therefore, we tested a postoperative model, excluding pN1. The postoperative factors, including pT, GG, SVI, pN and LVI, identified in this study have already been incorporated into nomograms as predictors of postoperative outcomes of RP,^15^ although pCC/IDCP has not been reported. O'Brein et al. developed an intriguing model that incorporated pIDCP into a nomogram predicting 3 year BCR after RP, but did not include pCC.^19^ Additionally, in an interesting report using RP specimens, Spratt et al. demonstrated that Decipher, a 22‐gene genomic classifier, independently predicted metastasis in men with persistent PSA after RP.^20^ Thus, we identified pCC/IDCP as a new postoperative factor associated with persistent PSA levels.

Based on the results of the analysis, three persistent PSA prediction models were established. Intriguingly, our model with preoperative factors showed areas under the receiver operating characteristic curve values that approximated those of the model with postoperative factors for predicting persistent PSA levels. For all the three models, the percentage of persistent PSA values after RARP was the highest in the high‐risk group. Using our predictive models, both early adjuvant and neoadjuvant therapies may be considered for patients with persistent PSA levels after RARP to improve prognosis.

Various aspects of the CC/IDCP and RP have been reported, and their relationships have become clear. Osiecki et al. reported that pCC/IDCP in RP specimens was associated with EPE (pooled OR = 2.55), SVI (pooled OR = 4.27), lymph node metastasis (pooled OR = 6.47), BCR (pooled OR = 5.09) and distant metastasis or CSM (pooled OR = 9.84).^6^ All these indicators (EPE, SVI and lymph node metastasis) have been reported to be important predictors of persistent PSA after RP,^2^ suggesting that IDCP/CC represents micrometastasis or local progression and is associated with persistent PSA.

Although there are many studies on CC/IDCP based on RP specimens, there are only some interesting reports on the impact of CC/IDCP based on prostate biopsy tissue on RP outcomes.^8^, ^9^ Yu et al. discovered that adding bCC/IDCP to the conventional Cancer of Prostate Risk Assessment and National Comprehensive Cancer Network scores improved the prediction of recurrence‐free and event‐free survivals in patients with RP.^9^ Okubo et al. revealed that bCC/IDCP was an independent risk factor for high GG levels and lymph node metastasis in RP specimens.^8^ Moreover, the National Comprehensive Cancer Network guideline recommends a genetic germline test for intermediate‐risk PCa patients with bCC/IDCP at diagnosis.^21^ Our study also confirmed the importance of bCC/IDCP.

Recently, the molecular characteristics of CC/IDCP were characterized.^22^, ^23^, ^24^, ^25^ Patients with CC tumours are more likely to have deletions at specific chromosomal sites (6q, 8p and 10q) and increased *SPOP* and *ATM* mutations.^23^ In contrast, IDCP has distinct genomic profiles, including frequent *TMPRSS2‐ERG* fusion, loss of *PTEN*, *RB1* and *TP53*.^24^ Chua et al. demonstrated that CC/IDCP PCa was associated with a PCa “nimbosus,” featuring genomic instability, hypoxia and expression of *SChLAP1*,^22^ resulting in poor outcomes. When these molecular biological conditions occur simultaneously, the rates of PSA relapse, distant metastasis occur and persistent PSA increase, leading to persistent PSA after RP.^22^ Therefore, we believe that pCC/IDCP, suggesting the possibility of micrometastases, was a significant factor in the analysis of Model 2, except for pN1 (a very strong predictive factor for persistent PSA).

This study has several limitations, including its relatively small sample size and retrospective design. First, the biopsy and pathology specimens were evaluated by experienced genitourinary pathologists at each institution; however, no central pathology review was performed. In our study, the CC/IDCP concordance rate between biopsy and final histological examination was lower than previous reports.^26^, ^27^ This could be due to sample errors, pathological interpretations or other reasons. Second, it is unclear whether persistent PSA levels after RARP are caused by residual benign prostate or residual cancer tissue. Third, although there are no uniform standards for persistent PSA levels and the timing of determination, a PSA half‐life of 3.15 days suggests that PSA levels of <50 ng/mL should become undetectable within 4 weeks.^3^ The Japanese Urological Association criteria (serum PSA levels had not decreased to <0.2 ng/mL at 1 month postoperatively and thereafter had never decrease to <0.2 ng/mL) were used for this cohort. The number of prostate biopsy cores varied, and multiple surgeons performed RARP. However, our new prognostic models, with the addition of CC/IDCP, were effective in predicting persistent PSA levels, particularly in models with preoperative factors. Further investigations are required to confirm these results.

bCC/IDCP predicted persistent PSA after RARP in the overall population, while pCC/IDCP predicted persistent PSA only when the pN1 population was excluded. This could be useful in predicting worse outcomes in susceptible patients.

## AUTHOR CONTRIBUTIONS

**Takeshi Sasaki:** Data collection and management; data analysis; manuscript writing and editing. **Ikuo Kobayashi:** Data collection and management. **Katsunori Uchida:** Data collection and management. **Shinichiro Higashi:** Data collection and management. **Satoru Masui:** Data collection and management. **Kouhei Nishikawa:** Data collection and management. **Toyonori Tsuzuki:** Protocol and project development; supervision. **Masatoshi Watanabe:** Protocol and project development; supervision. **Naoto Sassa:** Protocol and project development; supervision. **Takahiro Inoue:** Protocol and project development; data management; manuscript writing and editing.

## CONFLICT OF INTEREST STATEMENT

The authors have nothing to disclose.

## Supporting information


**Table S1 A.** Univariate and multivariate analysis of pre‐operative factors predicting pN1 after RARP with lymph node dissection (N = 567).
**B**. Univariate and multivariate analysis of post‐operative factors predicting pN1 after RARP with lymph node dissection (N = 567).

**Figure S1.** Kaplan–Meier analysis of MFS and CRPC‐FS in patients with and without persistent PSA levels after RARP. A. The three‐year MFS rates were 89.9% and 98.8% for PCa patients with and without persistent PSA levels, respectively *(P* < 0.001). B. The three‐year CRPC‐FS rates were 93.0% and 99.1% for PCa patients with and without persistent PSA levels, respectively (*P* = 0.003). MFS, metastasis‐free survival; CRPC‐FS, castration‐resistant prostate cancer‐free survival; RARP, robot‐associated radical prostatectomy
