# Supplementary material for: Cribriform pattern 4/intraductal carcinoma of the prostate and persistent prostate‐specific antigen after radical prostatectomy
Source: BJUI Compass. 2024 May 15;5(7):709–17. doi: 10.1002/bco2.367 (PMC11250726; doi:10.1002/bco2.367)
Supplement: Supplementary file 1 — Table S1 A. Univariate and multivariate analysis of pre‐operative factors predicting pN1 after RARP with lymph node dissection (N = 567). B. Univariate and multivariate analysis of post‐operative factors predicting pN1 after RARP with lymph node dissection (N = 567). [file BCO2-5-709-s001.docx]

| **Supplemental Table 1A**. Univariate and multivariate analysis of pre-operative factors predicting pN1 after RARP with lymph node dissection (N = 567). | | |
| --- | --- | --- |
|  |  |  |
| Factors | Univariate | Multivariate |
|  | Odds Ratio [95%CI]  (*P* value) | Odds Ratio [95%CI]  (*P* value) |
| Age (years) at surgery  (< 68 vs ≥ 68) | 1.17 [0.52 – 2.64]  (*P* = 0.693) | － |
| PSA levels (ng/mL) at surgery  (≥ 20 vs < 20) | 3.62 [1.28 – 10.22]  (*P* = 0.015) | － |
| PSAD (ng/mL/cm^3^)  (≥ 0.5 vs < 0.5) | 2.56 [1.12 – 5.85]  (*P* = 0.026) | － |
| Percentage of positive cancer cores (%)  (≥ 50 vs < 50) | 3.64 [1.62 – 8.18]  (*P* = 0.002) | － |
| Clinical T stage  (≥ 3 vs < 3) | 9.11 [3.90 – 21.31]  (*P* < 0.001) | 5.36 [2.12 – 13.54]  (*P* < 0.001) |
| Biopsy primary Gleason score 5  (Yes vs No) | 7.46 [2.71 – 20.53]  (*P* < 0.001) | 4.93 [1.59 – 15.33]  (*P* = 0.006) |
| Biopsy GG  (≥ 4 vs < 4) | 8.97 [3.03 – 26.52]  (*P* < 0.001) | 3.60 [1.07 – 12.02]  (*P* = 0.037) |
| Biopsy CC/IDCP  (Yes vs No) | 5.86 [2.55 – 13.48]  (*P* < 0.001) | 2.56 [1.00 – 6.52]  (*P* = 0.048) |
| Abbreviations |  |  |
| RARP, robot-assisted radical prostatectomy; PSA, prostate-specific antigen; PSAD, PSA density; GG, Grade Group; CC/IDCP, cribriform pattern 4 carcinoma/intraductal carcinoma of the prostate  All hazard ratios represent the ratios of the left-hand-side variables to the right-hand-side variables.  Only the variables found to be significant in univariate analysis (*P* < 0.05) were entered into the multivariate analysis. For the results of the multivariate analysis, only selected variables (*P* < 0.05) were listed in the table; variables that were not selected were hyphenated. | | |

| **Supplemental Table 1B**. Univariate and multivariate analysis of post-operative factors predicting pN1 after RARP with lymph node dissection (N = 567). | | |
| --- | --- | --- |
|  |  |  |
| Factors | Univariate | Multivariate |
|  | Odds Ratio [95%CI]  (*P* value) | Odds Ratio [95%CI]  (*P* value) |
| Pathological T stage  (≥ 3 vs < 3) | 7.91 [2.92 – 21.42]  (*P* < 0.001) | － |
| Pathological primary Gleason score 5　based on RARP specimens  (Yes vs No) | 4.72 [1.48 – 14.99]  (*P* = 0.008) | － |
| Pathological GG　based on RARP specimens  (≥ 4 vs < 4) | 10.95 [4.03 – 29.71]  (*P* < 0.001) | 3.89 [1.29 – 11.69]  (*P* = 0.015) |
| Pathological CC/IDCP　based on RARP specimens  (Yes vs No) | 9.41 [3.18 – 27.83]  (*P* < 0.001) | 3.93 [1.22 – 12.70]  (*P* = 0.022) |
| Nerve sparing  (Unilateral or bilateral vs None) | 0.29 [0.11 – 0.80]  (*P* = 0.017) | － |
| Surgical margins  (Positive vs Negative) | 4.17 [1.80 – 9.63]  (*P* < 0.001) | 3.27 [1.30 – 8.22]  (*P* = 0.011) |
| EPE  (Positive vs Negative or NA) | 10.64 [3.60 – 31.48]  (*P* < 0.001) | － |
| SV invasion  (Positive vs Negative) | 12.25 [5.28 – 28.40]  (*P* < 0.001) | － |
| LVI  (Positive vs Negative) | 13.55 [5.80 – 31.65]  (*P* < 0.001) | 6.67 [2.64 – 16.86]  (*P* < 0.001) |
| Abbreviations |  |  |
| PSA, Prostatic specific antigen; RARP, robot-assisted radical prostatectomy; GG, Grade Group;　CC/IDCP, cribriform pattern 4 carcinoma/intraductal carcinoma of the prostate; EPE, Extraprostatic extension; NA; not available; LVI, lymphovascular invasion; SV, seminal vesicle.  All hazard ratios represent the ratios of the left-hand-side variables to the right-hand-side variables.  Only the variables found to be significant in univariate analysis (*P* < 0.05) were entered into the multivariate analysis. For the results of the multivariate analysis, only selected variables (*P* < 0.05) were listed in the table; variables that were not selected were hyphenated. | | |
