# Supplementary material for: Cribriform pattern 4/intraductal carcinoma of the prostate and persistent prostate‐specific antigen after radical prostatectomy
Source: BJUI Compass. 2024 May 15;5(7):709–17. doi: 10.1002/bco2.367 (PMC11250726; doi:10.1002/bco2.367)
Supplement: Supplementary file 2 — Figure S1. Kaplan–Meier analysis of MFS and CRPC‐FS in patients with and without persistent PSA levels after RARP. A. The three‐year MFS rates were 89.9% and 98.8% for PCa patients with and without persistent PSA levels, respectively (P < 0.001). B. The three‐year CRPC‐FS rates were 93.0% and 99.1% for PCa patients with and without persistent PSA levels, respectively (P = 0.003). MFS, metastasis‐free survival; CRPC‐FS, castration‐resistant prostate cancer‐free survival; RARP, robot‐associated radical prostatectomy [file BCO2-5-709-s002.pdf]

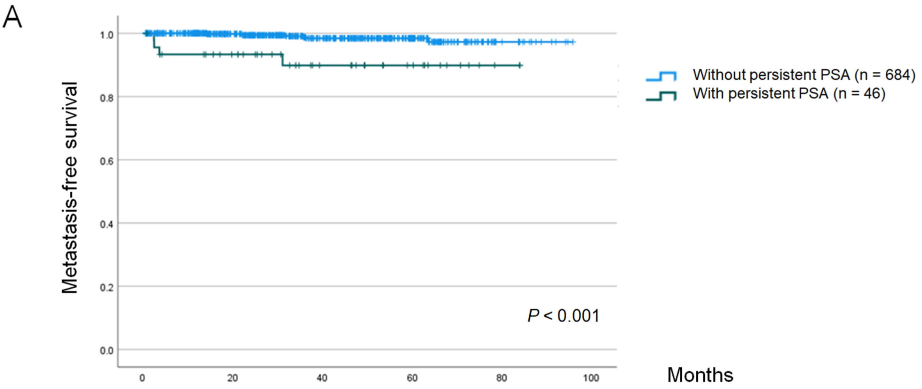


| No. of patients at risks | | | | | |
|---|---|---|---|---|---|
| Without persistent PSA | 684 | 479 | 280 | 113 | 16 |
| With persistent PSA | 46 | 35 | 20 | 12 | 2 |

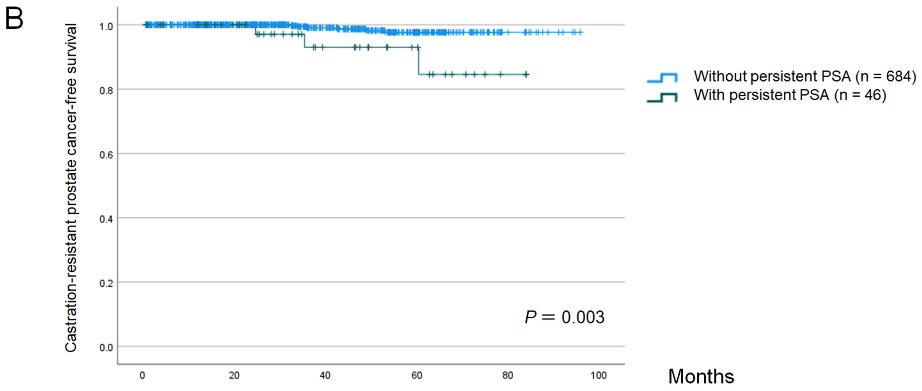


| No. of patients at risks | | | | | |
|---|---|---|---|---|---|
| Without persistent PSA | 684 | 480 | 281 | 111 | 16 |
| With persistent PSA | 46 | 37 | 20 | 12 | 2 |
